# Synthesis and Biological Evaluation of Benzochromenopyrimidinones as Cholinesterase Inhibitors and Potent Antioxidant, Non-Hepatotoxic Agents for Alzheimer’s Disease

**DOI:** 10.3390/molecules21050634

**Published:** 2016-05-14

**Authors:** Youssef Dgachi, Oscar M. Bautista-Aguilera, Mohamed Benchekroun, Hélène Martin, Alexandre Bonet, Damijan Knez, Justyna Godyń, Barbara Malawska, Stanislav Gobec, Mourad Chioua, Jana Janockova, Ondrej Soukup, Fakher Chabchoub, José Marco-Contelles, Lhassane Ismaili

**Affiliations:** 1 Laboratory of Applied Chemistry, Heterocycles, Lipids and Polymers, Faculty of Sciences of Sfax, University of Sfax, B.P. 802, Sfax 3000, Tunisia; youssefdgachi@gmail.com; 2 Laboratoire de Chimie Organique et Thérapeutique, Neurosciences Intégratives et Cliniques, EA 481, UFR SMP, Univ. Franche-Comté, Univ. Bourgogne Franche-Comté, 19, rue Ambroise Paré, Besançon F-25000, France; osmabaga@gmail.com (O.M.B.-A.); mohamed.benchekroun@outlook.com (M.B.); 3 Laboratory of Cell Toxicology, EA 4267, University of Bourgogne Franche-Comté, 19 rue Ambroise Paré, Besançon Cedex 25030, France; helene.martin@univ-fcomte.fr (H.M.); alexandre.bonet@univ-fcomte.fr (A.B.); 4 Faculty of Pharmacy, University of Ljubljana, Aškerčeva 7, Ljubljana 1000, Slovenia; damijan.knez@ffa.uni-lj.si (D.K.); stanislav.gobec@ffa.uni-lj.si (S.G.); 5 Department of Physicochemical Drug Analysis, Jagiellonian University Medical College, Medyczna 9 Street, Krakow 30-688, Poland; justyna.godyn@uj.edu.pl (J.G.); mfmalaws@cyf-kr.edu.pl (B.M.); 6 Laboratory of Medicinal Chemistry (IQOG, CSIC) C/Juan de la Cierva 3, Madrid 28006, Spain; mchioua@gmail.com; 7 Biomedical Research Center, University Hospital Hradec Kralove, 500 05 Hradec Králove, Czech Republic; jana.janockova@fnhk.cz (J.J.); soukup.ondrej@fnhk.cz (O.S.)

**Keywords:** Alzheimer’s disease, quinazolinones, multicomponent reactions, multitarget-directed ligands, antioxidants, cholinesterase inhibitors, hepatotoxicity

## Abstract

We report herein the straightforward two-step synthesis and biological assessment of novel racemic benzochromenopyrimidinones as non-hepatotoxic, acetylcholinesterase inhibitors with antioxidative properties. Among them, compound **3Bb** displayed a mixed-type inhibition of human acetylcholinesterase (IC_50_ = 1.28 ± 0.03 μM), good antioxidant activity, and also proved to be non-hepatotoxic on human HepG2 cell line.

## 1. Introduction

Alzheimer’s disease (AD) has emerged as the main cause of memory and cognitive deficiency in aged persons. The Alzheimer’s disease International (ADI) Association report from 2015 estimates that over 46 million people are currently affected by dementia. Given the epidemic expansion of AD, this number is expected to drastically increase in the future, reaching 131.5 million cases by 2050 [1]. Many efforts have been devoted to comprehend the complex etiology of AD, yet certain aspects of the pathogenesis are still not well understood. Nevertheless, several histopathologic features have been clearly identified in AD patients such as intracellular neurofibrillary tangles, composed of hyperphosphorylated tau protein and toxic amyloid plaques of aggregated *β*-amyloid (A*β*) peptide. Moreover, different levels of neuronal loss have been observed in the *Locus coeruleus* [2], *Nucleus basalis* [3] and *Substantia nigra* [4] brain areas, leading to substantial perturbations in several neurotransmission systems such as the cholinergic, serotoninergic, GABAergic, glutamatergic, noradrenergic or dopaminergic system.

Oxidative stress holds a fundamental position in the onset and development of AD. Several studies have confirmed that the constant accumulation of reactive oxygen species and reactive nitrogen species leads inevitably to serious oxidative damage in neuronal tissues [5]. This oxidative stress can be triggered by different underlying factors such as mitochondrial dysfunction [6], loss of metal homeostasis (e.g., Cu^2+^, Fe^2+^, Zn^2+^), the involvement of the later ions in Aβ aggregation [7], and neuroinflammation [8]. Globally, there is unanimity to consider these different biological events all at once, and address the unmet need for an efficient anti-AD agent.

In terms of medication use, the currently marketed drugs are mainly inhibitors of cholinesterases (ChEIs) (acetylcholinesterase, AChE; and butyrylcholinesterase, BuChE). These are donepezil, rivastigmine, and galantamine [9], which enhance the levels of neurotransmitter acetylcholine in the synaptic cleft. The only drug with a different mechanism of action is memantine, a *N*-methyl-d-aspartate receptor antagonist [10]. One of the first marketed ChEI, tacrine, was rapidly withdrawn, principally because of its hepatotoxicity [11]. Globally, the therapeutic efficacy of pure ChEIs may be brought into question since only scarce improvements in memory and cognitive functions have been reached in AD patients, with no signs of clear reversal of the disease. Therefore, the design of new multitarget-directed ligands represents one of the most promising approaches for the development of new disease-modifying agents for AD therapy [12,13,14]. Indeed, compounds capable to simultaneously modulate various biological systems in relation with AD pathogenesis might be a winning strategy in the future by furnishing fine-tuned drug candidates for the clinics.

**Figure 1:**
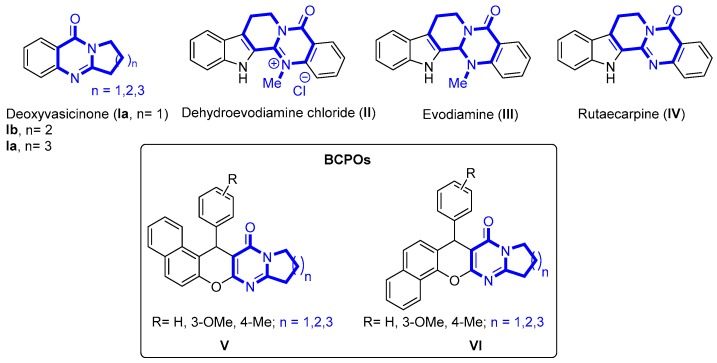
The quinazolinone alkaloids **Ia**–**IV** and designed BCPOs **V** and **VI**.

Several series of quinazolinone derivatives have already been developed, inspired by naturally occurring alkaloids deoxyvasicinone (**Ia**), dehydroevodiamine chloride (**II**), evodiamine (**III**) and rutaecarpine (**IV**) (Figure 1), with promising inhibition of ChEs [15,16,17]. Further SAR investigations were done and revealed that some new carbamate analogues of evodiamine were selective butyrylcholinesterase inhibitors (BuChEIs), potent antioxidants and neuroprotective agents against glutamate-induced oxidative stress in HT-22 cells [16].

In relation with these antecedents and with our earlier work [18,19], we applied a multicomponent reaction approach to further explore the chemical space based on the quinazolinone scaffold. Thus, we have designed new benzochromenopyrimidinones of type **V** and **VI** (abbreviated as BCPOs, Figure 1), where a benzochromene motif was fused to the pyrimidinone motif present in alkaloids **Ia**–**IV**. As a result, eighteen new BCPOs were synthesized and evaluated for their antioxidant activity, ChE inhibition, and their *in vitro* toxicity in liver HepG2. From these studies, we have identified compound **3Bb** as a promising derivative potentially useful in further AD drug discovery steps.

## 2. Results and Discussion

### 2.1. Synthesis

**Scheme 1:**
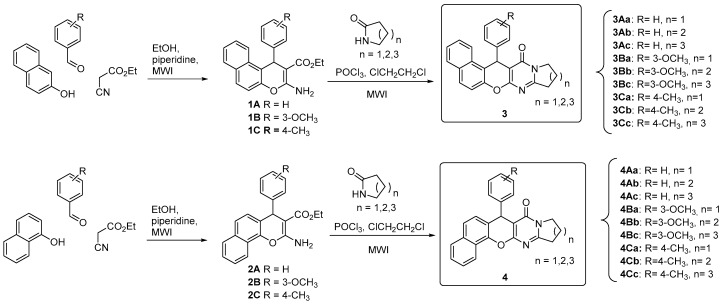
Synthesis of BCPOs **3** and **4**.

The synthesis of the target BCPOs **3** and **4** has been carried out in two steps, and good overall yields as outlined in Scheme 1. First, a microwave-assisted multicomponent reaction of ethyl cyanoacetate, selected aromatic aldehydes, and 2- or 1-naphthol, in the presence of a catalytic amount of piperidine in ethanol, at 80 °C, for 10 min, gave the corresponding ethyl 3-amino-1-phenyl-1*H*-benzo[*f*]chromene-2-carboxylates **1A**–**C** or ethyl 2-amino-4-phenyl-4*H*-benzo[*h*]chromene-3-carboxylates **2A**–**C**, respectively, in good yields (68%–90%). The second step was the condensation of adducts **1A**–**C** or **2A**–**C** with the appropriate commercial lactams, in the presence of phosphorus oxytrichloride in 1,2-dichloroethane, under microwave irradiation for 15 min at 80 °C, to give compounds **3** and **4** in high yields (70%–96%). All new compounds displayed satisfactory analytical and spectroscopic data correlating with their structure, and with the data reported in the literature for comparable molecules (see Experimental Section).

### 2.2. Evaluation of the Antioxidant Power

First of all, we evaluated the antioxidant activity of compounds **3** and **4** using the oxygen radical absorbance capacity by fluorescence (ORAC-FL) method [20,21]. Trolox was used as standard, fluorescein as fluorescent probe and 2,2′-azobis(2-amidinopropane) dihydrochloride (AAPH) as the peroxyl radical source. Ferulic acid was used as a positive control [22]. Data were expressed as Trolox equivalents (TE), and as shown in table 1, all compounds were able to scavenge the peroxyl radical with ORAC values ranging between 2.3 and 4.7 TE. The unsubstituted adducts **3Aa**–**c**, **4Aa**–**c** were found to be slightly less potent than the analogues bearing methoxy and methyl groups at the aromatic ring, with values ranging between 2.1 and 2.6 TE. However, when the phenyl moiety was substituted by a methoxy group, we globally observed a better antioxidant activity, compound **3Bb** being the most active (4.7 TE) and displaying enhanced antioxidant activity compared to ferulic acid (3.7 TE).

### 2.3. Evaluation of AChE and BuChE Inhibition

For the preliminary screening of the inhibitory potencies, *Electrophorus electricus* AChE (*Ee*AChE) and horse serum BuChE (*eq*BuChE) were used following the Ellman’s assay [23]. Tacrine, able to inhibit both ChEs, was selected as a control. First of all, compounds **3** and **4** were poor *eq*BuChEIs, and due to the limited solubility in the assay medium, only the percentage of inhibition at 10 µM was determined. However, these compounds exhibited encouraging inhibitory potencies against *Ee*AChE with IC_50_ values ranging from 30.5 to 518.4 nM. The most potent *Ee*AChEIs, in decreasing order, were compounds **3Bb**, **3Ab**, **3Cb** and **3Ba** with IC_50_ 30.5, 55.5, 55.9 and 60.7 nM values, respectively, showing activities comparable to that of tacrine (IC_50_ = 44.3 nM). Very interestingly, and for comparative purposes, related natural alkaloids **Ia** [24] or **II** [15], and synthetic compounds **Ib**,**c** [24] are poorer *Ee*AChEIs than BCPOs **3** and **4** (Table 1).

When examining the structure-activity relationships (SAR), we could observe that the four most active compounds (**3Ab**, **3Bb**, **3Cb** and **3Ba**) belong to the **V** family (Figure 1). Concerning the size of the saturated carbocyclic ring attached to the pyrimidinone moiety and considering the same substituent on the aromatic ring attached at the stereogenic center, the most potent AChEIs were compounds bearing a piperidine-fused ring (**3Ab**, **3Bb**, and **3Cb**) for the **V** family. For the **VI** type derivatives, no evident SAR could have been established. Finally, we can notice that BCPOs of type **V**, with a methoxy-substituted benzene ring had IC_50_ values for the inhibition of *Ee*AChE much higher than non-substituted analogues.

Based on these findings, we selected compounds **3Ab**, **3Bb**, **3Cb** and **3Ba** for the Aβ_1–42_ aggregation inhibition studies. Unfortunately, only compound **3Ab** showed a weak inhibition power (Supplementary Materials). Next, we investigated the ability of compounds **3Ab**, **3Bb**, **3Cb** and **3Ba** to inhibit human recombinant AChE (*h*AChE), and their liver toxicity.

### 2.4. Kinetic Study of the hAChE Inhibition by Compound ***3Bb***

As shown in Table 1, we found significantly lower inhibition for *h*AChE in comparison with *Ee*AChE, the IC_50_ values ranging from 1279 to 3657 nM. Compound **3Bb** was the most potent inhibitor with an IC_50_ value of 1279 nM.

**Figure 2:**
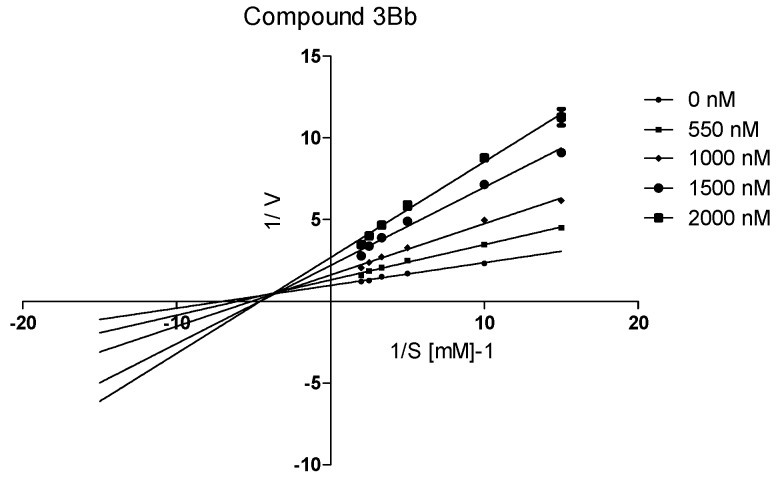
Lineweaver–Burk double reciprocal plot demonstrating mixed-type of *h*AChE inhibition by compound **3Bb**. S = acetylthiocholine; *V* = initial velocity rate.

To get insight into the mode of inhibition, the kinetic mechanism of *h*AChE inhibition by compound **3Bb** was investigated through classical Lineweaver-Burk double reciprocal plots. Analysis of this plot (Figure 2) showed the interception of the lines above the *x*-axis indicating that **3Bb** is able to interact with both the free and acylated enzyme, and therefore behaves as mixed-type inhibitor of *h*AChE. The inhibitor dissociation constants *K_i_* (dissociation constant for the enzyme-inhibitor complex) and *K’*_i_ (dissociation constant for the enzyme-inhibitor-substrate complex) were estimated and were 0.38 and 1.12 μM, respectively.

### 2.5. In Vitro Toxicity of Compounds ***3Ab**, **3Bb**, **3Cb*** and ***3Ba*** in HepG2 Cells

A prerequisite for any effective lead drug is to keep its cytotoxicity at the lowest possible level. In this regard, we submitted the four most promising compounds (**3Ab**, **3Bb**, **3Cb** and **3Ba**) to an *in vitro* toxicologic evaluation (MTT assay) using human hepatocellular carcinoma cell line (HepG2) which represents a good probe to evaluate hepatotoxic effects. As shown in Table 2, tacrine was safe up to 100 µM but significantly decreased cell viability above 300 μM. The four tested BCPOs had no toxic effects on the HepG2 cells (Table 2) measured in concentrations up to 1000 μM [25] and could therefore be considered as non-hepatotoxic.

### 2.6. Blood Brain Barrier Penetration PAMPA Assay

Prediction of blood-brain barrier (BBB) penetration is summarized in Table 3. Compound **3Ab** showed the highest probability to cross the BBB via passive diffusion. Compound **3Ba** seems to be also central nervous systems (CNS) available according to the results obtained, however Pe value is on the lower limit for permeable compounds. Compounds **3Bb** and **3Cb** were not satisfactorily distinguished. Whereas Pe value for the compound **3Bb** fails into the uncertain interval, the value for the compound **3Cb** was not determined due to the low solubility of the compound and therefore low UV/Vis absorption.

Data obtained for the new compounds correlate well with values for controls drugs, where CNS availability is known and also reported using the PAMPA assay [26,27]. Our data show high resemblance with previously reported results as well as with a general knowledge about the CNS availability for these drugs.

## 3. Materials and Methods

### 3.1. Chemistry Methods

Melting points were determined on a Kofler apparatus (Wagner Munz, München, Germany), and are uncorrected. Progress of the reactions was monitored with TLC using aluminium sheets with silica gel 60 F254 from Merck (Kenilworth, NJ, USA). IR spectra were recorded on a PARAGON FT-IR spectrometer (Perkin-Elmer, Waltham, MA, USA) covering field 400–4000 cm^−1^. ^1^H-NMR and ^13^C-NMR were recorded on a Bruker spectrometer (Bruker BioSpin, Fällanden, Switzerland) (^1^H-NMR at 300 MHz, ^13^C-NMR at 75 MHz) using CDCl_3_ or DMSO-*d*_6_ as solvents. The chemical shifts are reported in parts per million (ppm), using tetramethylsilane (TMS) as internal reference. The multiplicities of the signals are indicated by the following abbreviations: s, singlet; d, doublet; t, triplet; q, quadruplet; and m, multiplet coupling constants are expressed in Hz. Elemental analysis were performed on Flash EA 1112 Thermo Finnigan, (Thermo scientific, Waltham, MA, USA). The microwave assisted reactions were carried out in synthesis microwave (Anton Paar 300, Peseux, Switzerland) with a maximum power of 300 W.

#### 3.1.1. General Procedure for the Compounds **1A**–**C** and **2A**–**C**

A mixture of appropriate aromatic aldehyde (0.01 mol), ethyl cyanoacetate (0.01 mol), 1-naphthol (or 2-naphthol) (0.01 mol) in ethanol (20 mL) in the presence of piperidine (0.2 equiv) was irradiated for 10 min in a sealed tube. The irradiation was programed to maintain a constant temperature (80 °C, 150 W). The obtained precipitate was filtered, washed with cold ethanol and dried, to give the desired compounds.

*Ethyl 3-amino-1-phenyl-1H-benzo[f]chromene-2-carboxylate* (**1A**) [27]: Yield 90%; mp 168–170 °C; IR (KBr) ν_max_ 3300–3438, 1685 cm**^−^**^1^; ^1^H-NMR (DMSO-*d*_6_) 1.23–1.28 (m, 3H), 4.08–4.17 (m, 2H), 5.51 (s, 1H), 7.00–8.02 (m, 11H, arom.), 7.67 (s, 2H, NH_2_); ^13^C-NMR (DMSO-*d*_6_) 14.9, 37.0, 59.3, 78.2, 117.2, 119.3, 123.6, 125.2, 126.4, 127.5, 128.2, 128.5, 129.0, 129.4, 130.7, 131.2, 147.2, 147.3, 160.9, 168.6.

*Ethyl 3-amino-1-(3-methoxyphenyl)-1H-benzo[f]chromene-2-carboxylate* (**1B**): Yield 73%; mp 168–170 °C; IR (KBr) ν_max_ 3339–3425, 1690 cm**^−^**^1^; ^1^H-NMR (DMSO-*d*_6_) 1.14–1.20 (m, 3H), 3.80 (s, 3H), 4.01–4.12 (m, 2H), 5.82 (s, 1H), 6.72–8.25 (m, 10H, arom.), 7.68 (s, 2H, NH_2_); ^13^C-NMR (DMSO-*d*_6_) 14.7, 31.3, 55.8, 59.1, 77.5, 111.6,117.1, 119.5, 120.8, 123.7, 125.0, 127.3, 127.7, 128.9, 128.9, 130.5, 131.0, 131.3, 135.5, 147.3, 156.1, 161.4, 169.0.

*Ethyl 3-amino-1-p-methylphenyl-1H-benzo[f]chromene-2-carboxylate* (**1C**) [28]: Yield 75%; mp 194–196 °C; IR (KBr) ν_max_ 3320–3440 (NH_2_), 1687 (CO) cm**^−^**^1^; ^1^H-NMR (DMSO-*d*_6_) 1.24–1.31 (m, 3H), 2.10 (s, 3H), 4.09–4.23 (m, 2H), 5.49 (s, 1H), 6.93–8.01 (m, 10H, arom.), 7.68 (s, 2H, NH_2_); ^13^C-NMR (DMSO-*d*_6_) 14.9, 20.9, 36.6, 59.3, 78.3, 117.1, 119.4, 123.6, 125.2, 127.4, 128.0, 129.0, 129.1, 129.3, 130.8, 131.2, 135.3, 144.4, 147.2, 160.9, 168.7.

*Ethyl 2-amino-4-phenyl-4H-benzo[h]chromene-3-carboxylate* (**2A**) [29]: Yield 88%; mp 160-162 °C; IR (KBr) ν_max_ 3372–3260, 1692 cm**^−^**^1^; ^1^H-NMR (DMSO-*d*_6_) 1.10–1.18 (m, 3H), 4.01–4.11 (m, 2H), 5.04 (s, 1H), 7.04–8.33 (m, 11H, arom.), 7.64 (s, 2H, NH_2_); ^13^C-NMR (DMSO-*d*_6_) 14.7, 40.5, 59.0, 76.8, 121.1, 121.4, 123.2, 124.1, 126.4, 126.9, 126.9, 127.0, 127.7, 128.1, 128.6, 132.9, 143.2, 148.2, 161.2, 168.7.

*Ethyl 2-amino-4-m-methoxyphényl-4H-benzo[h] chromene-3-carboxylate* (**2B**): Yield 75%; mp 156–158 °C; IR (KBr) ν_max_ 3393–3280, 1663 cm**^−^**^1^; ^1^H-NMR (DMSO-*d*_6_) 1.09–1.18 (m, 3H), 3.68 (s, 3H), 4.02–4.14 (m, 2H), 5.01 (s, 1H), 6.67–8.32 (m, 10H, arom.), 7.63 (s, 2H, NH_2_); ^13^C-NMR (DMSO- *d*_6_) 14.2, 40.0, 54.8, 58.6, 76.2, 110.7, 113.5, 119.5, 120.6, 120.8, 122.7, 123.6, 126.4, 126.4, 126.5, 127.6,129.2, 132.4, 142.8, 149.3, 159.0, 160.8, 168.2.

*Ethyl-2-amino-4-p-methylphenyl-4H-benzo[h]chromene-3-carboxylate* (**2C**) [29]: Yield 68%; mp 158–160 °C; IR (KBr) ν_max_ 3315–3452, 1672 cm**^−^**^1^; ^1^H-NMR (DMSO-*d*_6_) 1.10–1.17 (m, 3H), 2.28 (s, 3H), 4.00–4.09 (m, 2H), 4.98 (s, 1H), 6.98–8.29 (m, 10H, arom.), 7.62 (s, 2H, NH_2_); ^13^C-NMR (DMSO-*d*_6_) 14.7, 20.9, 40.8, 59.0, 76.9, 121.2, 121.8, 123.3, 124.1, 126.8, 126.9, 127.0, 127.6, 128.0, 129.2,132.9, 135.4, 143.2, 145.3, 161.2, 168.7.

#### 3.1.2. General Procedure for the Synthesis of Benzochromenopyrimidinones (BCPOs)

POCl_3_ (0.14 mL, 0.23 g, 1.5 equiv) was added dropwise to a mixture of the corresponding ethyl aminobenzochromene-2-carboxylate and the appropriate lactam (1.5 equiv) in 1,2-dichloroethane (20 mL). After microwave irradiation, approximately 80% of the solvent was evaporated and water (10 mL) was added. The solution was basified with 20% aqueous NaOH, then the mixture was extracted with CH_2_Cl_2_, washed with water (20 mL), and dried over MgSO_4_. The solvent was evaporated, the solid obtained was washed with ether and filtered to give benzochromenopyrimidinones **3** and **4**.

*14-Phenyl-10,11-dihydro-14H-benzo[5,6]chromeno[2,3-d]pyrrolo[1,2-a]pyrimidin-13(9H)-one* (**3Aa**): Yield 80%; mp ˃ 260 °C; IR (KBr) ν_max_ 1667, 1587 cm^−1^; ^1^H-NMR (CDCl_3_) 2.18–2.22 (m, 2H, H10), 2.28–2.36 (m, 2H, H9), 3.20–3.29 (m, 2H, H11), 5.31 (s, 1H, H14), 6.64 (d, *J* = 7.8 Hz 1H, H6), 7.19–7.48 (m, 7H, H2, H3, H2′,H5′,H3′,H6′,H4′), 7.80 (d, *J* = 8.1 Hz, 2H, H4, H5), 7.96 (d, *J* = 8.4 Hz,1H,H1); ^13^C-NMR (CDCl_3_) 18.6 (CH_2_, C10), 32.0 (CH_2_, C9), 36.0 (CH, C14), 46.6 (CH, C11), 100.8 (C, C13a), 115.9 (CH, C6), 116.9 (CH, C1), 123.1 (CH, C3), 124.4 (CH, C2), 126.1 (CH, C4), 126.3 (CH, C5), 126.6 (CH, C4′), 127.8 (2 CH, C2′, C6′), 127.9 (2 CH, C3′, C5′), 128.8 (C, C14b), 130.6 (C, C14a), 131.0 (C, C4a), 143.3 (C, C1′), 147.8 (C, C6a), 160.5 (C, C8a), 160.9 (C, C7a), 162.1 (C, C13). Anal. Calcd. for C_24_H_18_N_2_O_2_: C, 78.67; H, 4.95; N, 7.65. Found: C, 78.61; H, 4.98; N, 7.69.

*15-Phenyl-10,11,12,15-tetrahydro-9H-benzo[5,6]chromeno[2,3-d]pyrido[1,2-a]pyrimidin-14-one* (**3Ab**): Yield 96%; mp 236 °C; IR (KBr) ν_max_ 1669, 1587 cm^−1^; ^1^H-NMR (CDCl_3_) 1.83–1.94 (m, 4H, H11, H10), 2.91–2.94 (m,H9, 2H), 3.87–3.93 (m, H12, 2H), 5.76 (s, H15, 1H), 7.07 (d, *J* = 7.8 Hz, H6, 1H), 7.16–7.52 (m, H2, H3, H5, H2′,H5′,H3′,H6′,H4′, 8H), 7.97 d (*J* = 8.4 Hz, H1, H4, 2H); ^13^C-NMR (CDCl_3_) 18.2 (CH_2_, C10), 20.9 (CH_2_, C11), 30.9 (CH_2_, C9), 35.9 (CH, C15), 42.4 (CH, C12), 99.6 (CH, C14a), 116.4 (C, C6), 117.3 (CH, C1), 123.3 (CH, C3), 124.9 (CH, C2), 126.4 (CH, C4), 127.1 (CH, C5), 128.1 (CH,C4′), 128.2 (2 CH, C2′, C6′), 128.5 (2 CH, C3′, C5′), 129.4 (C, C15b), 130.4 (C, C15a), 130.9 (C, C4a), 144.2 (C, C1′), 147.7 (C, C6a), 158.9 (C, C8a), 159.4 (C, C7a), 161.4 (C,C14). Anal. Calcd. for C_25_H_20_N_2_O_2_: C, 78.93; H, 5.30; N, 7.36. Found: C, 78.92; H, 5.32; N, 7.32.

*16-Phenyl-10,11,12,13,-tetrahydro-16H-benzo[5,6]chromeno[2′,3′,4,5]pyrimido[1,2-a]azepin-15(9H)-one (***3Ac**): Yield 85%; mp 242 °C; IR (KBr) ν_max_ 1660, 1589 cm^−1^; ^1^H-NMR (CDCl_3_) 1.78–1.83 (m, H12, H11, H10, H9, 8H), 2.97–2.99 (m, H13, 2H), 5.89 (s, H16, 1H), 7.09 d (*J* = 7.8 Hz, H6, 1H), 7.19–7.48 m (m, H2, H3, H2′, H5′, H3′, H6′,H4, 7H), 7.81 (d, *J* = 8.1 Hz, H4, H5, 2H), 7.96 (d, *J* = 8.4 Hz,H1, 1H); ^13^C-NMR (CDCl_3_) 24.0 (CH_2_,C10), 26.7 (CH_2_,C11), 29.1 (CH_2_, C12), 36.4 (CH_2_,C16), 36.8 (CH, C9), 42.6 (CH, C13), 100.6 (CH,C15a), 116.2 (C,C6), 117.0 (CH, C1), 123.2 (CH,C3), 124.3(CH,C2), 126.0 (CH,C4), 126.5 (CH, C5), 127.9 (2 CH, C2′, C6′), 128.0 (2 CH, C3′, C5′), 128.4 (CH, C4′), 128.7 (C, C16b), 130.6 (C, C16a), 130.9 (C, C4a), 143.4 (C, C1′), 147.7 (C, C6a), 158.8 (C, C8a), 161.6 (C, C7a), 162.5 (C, C15). Anal. Calcd. for C_26_H_22_N_2_O_2_: C, 79.17; H, 5.62; N, 7.10. Found: C, 79.25; H, 5.60; N, 7.17.

*14-(3′Methoxyphenyl)-10,11-dihydro-14H-benzo[5,6]chromeno[2,3-d]pyrrolo[1,2-a]pyrimidin-13(9H)-one* (**3Ba**): Yield 81%; mp 211 °C; IR (KBr) ν_max_1660, 1591cm^−1^; ^1^H-NMR (CDCl_3_) 2.22–2.27 (m, H10, 2H), 3.01–3.14 (m, H9, 2H), 3.71 (s, OCH_3_, 3H), 4.13–4.17 (m, H11, 2H), 5.92 (s, H14, 1H), 6.66 (d, *J* = 7.8 Hz, H6, 1H), 6.99–7.48 (m, H2, H3, H2′,H5′, H6′, H4′, 6H), 7.82 (d, *J* = 8.4 Hz, H4, H5, 2H), 7.99 (d, *J* = 8.1 Hz, H1, 1H); ^13^C-NMR (CDCl_3_) 18.6 (CH, C10), 31.9 (CH, C9), 35.9 (CH, C14), 46.5 (CH, C11), 55.0 (OCH_3_), 100.7 (C, C13a), 111.3 (CH, C4′), 114.0 (CH, C2′), 115.9 (CH,C5), 116.9 (CH,C6′), 120.4 (C, C14a), 123.1 (CH, C1), 124.4 (CH, C3), 126.5 (CH, C2), 127.9 (CH, C6), 128.7 (CH, C4), 128.8 (C, C4a), 130.6 (CH, C5′), 131.0 (C, C14b), 144.9 (C, C1′), 147.8 (C, C6a), 158.1 (C, C8a), 160.6 (C, C7a), 161.0 (C, C13), 162.0 (C, C3′). Anal. Calcd. for C_25_H_20_N_2_O_3_: C, 75.74; H, 5.09; N, 7.07. Found: C, 75.69; H, 5.12; N, 7.11.

*15-(3′-Methoxyphenyl)-10,11,12,15-tetrahydro-9H-benzo[5,6]chromeno[2,3-d]pyrido[1,2-a]pyrimidin-14-one* (**3Bb**): Yield 90%; mp 212 °C; IR (KBr) ν_max_ 1658, 1583 cm^−1^; ^1^H-NMR (CDCl_3_) 1.85–1.94 (m, H9, H10, H11, 6H), 2.86–2.94 (m, H12, 2H), 3.71 (s, OCH_3_, 3H), 5.89 (s, H15, 1H), 6.65 (d, *J* = 7.8 Hz, H6, 1H), 6.99–7.46 (m, H2, H3, H2′, H5′, H6′, H4′, 6H), 7.80 (d, *J* = 8.4 Hz, H4, H5, 2H), 7.98 (d, *J* = 8.1 Hz,H1, 1H); ^13^C-NMR (CDCl_3_) 19.0 (CH,C10), 21.8 (CH, C11), 31.5 (CH, C9), 36.6 (CH, C15), 42.9 (CH, C12), 55.0 (C, OCH_3_), 100.8 (C,C14a), 114.6 (CH, C2′), 116.6 (CH, C5), 117.1 (CH, C4′), 117.5 (CH, C6′), 121.0 (C, C15a), 123.7 (CH, C1), 124.8 (CH, C3), 127.0 (CH, C2), 128.4 (CH, C6), 129.1 (CH, C4), 129.3 (C, C4a), 131.0 (C, C15b), 131.2 (CH, C5′), 145.5 (C, C1′), 148.2 (C, C6a), 158.5 (C,C8a), 159.0 (C, C7a), 159.5 (C, C14), 162.4 (C, C3′). Anal. Calcd. or C_26_H_22_N_2_O_3_: C, 76.08; H, 5.40; N, 6.82. Found: C, 76.13; H, 5.36; N, 6.85.

*16-(3′-Methoxyphenyl)-10,11,12,13-tetrahydro-16H-benzo[5,6]chromeno[2′,3′,4,5]pyrimido[1,2-a]azepin-15(9H)-one* (**3Bc**): Yield 82%; mp 206 °C; IR (KBr) ν_max_ 1662, 1585 cm^−1^; ^1^H-NMR (CDCl_3_) 1.79–2.33 (m, H9, H10, H11, H12, H8), 2.92–2.99 (m, H13, 2H), 3.71 (s, OCH_3_, 3H), 5.87 (s, H16, 1H), 6.65 (d, *J* = 7.8 Hz, H6, 1H), 6.84–7.49 (m, H2, H3, H2′,H5′, H6′, H4′, 6H), 7.80 (d, *J* = 8.4 Hz, H4,H5, 2H), 7.98 (d, *J* = 8.1 Hz, H1, 1H); ^13^C-NMR (CDCl_3_) 24.5 (CH, C10), 27.2 (CH, C11), 29.7 (CH, C12), 36.9 (CH, C16), 37.4 (CH, C9), 43.2 (CH, C13), 55.0 (OCH_3_), 101.0 (C, C15a), 111.6 (C, C4′), 114.6 (CH, C2′), 116.6 (CH, C5), 117.5 (CH, C6′), 121.1 (C, C16a), 123.7 (CH, C1), 124.9 (CH, C3), 127.0 (CH, C2), 128.4 (CH, C6), 129.2 (CH, C4), 129.3 (C, C4a), 131.1 (CH, C5′), 131.4 (C, C16b), 145.4 (C, C1′), 148.1 (C, C6a), 159.3 (C, C8a), 159.5 (C, C7a), 162.1 (C, C15), 163.5 (C, C3′). Anal. Calcd. for C_27_H_24_N_2_O_3_: C, 76.40; H, 5.70; N, 6.60. Found: C, 76.36; H, 5.73; N, 6.64.

*14-(4′-Methylphenyl)-10,11-dihydro-14H-benzo[5,6]chromeno[2,3-d]pyrrolo[1,2-a]pyrimidin-13(9H)-one* (**3Ca**): Yield 82%; mp > 260 °C; IR (KBr) ν_max_ 1661, 1594 cm^−1^; ^1^H-NMR (CDCl_3_) 1.21–1.25 (m, H10, 2H), 2.2 (s, CH_3_, 3H), 3.06–3.12 (m, H9, 2H), 4.05–4.11 (m, H11, 2H), 5.90 (s, H14, 1H), 7.05 (d, *J* = 8.7 Hz, H3′, H5′, 2H), 7.28–7.47 (m, H2, H3, H4, H5, H6, 5H), 7.81 (d, *J* = 8.7 Hz, H2′, H6′, 2H),7.97 d (d, *J* = 8.1 Hz, H1, 1H); ^13^C-NMR (CDCl_3_) 19.1 (CH_2_, C10), 20.9 (CH_3_), 32.4 (CH, C9), 36.0 (CH, C14), 47.0 (CH, C11), 101.5 (C, C13a), 116.7 (CH, C6), 117.5 (CH, C1), 123.7 (CH, C3), 124.8 (CH, C2), 127.0 (CH, C4), 128.3 (CH, C5), 128.4 (2CH, C2′, C6′), 129.0 (2CH, C3′, C5′), 129.2 (C, C14b), 131.1 (C, C14a), 131.5 (C, C4a), 136.2 (C, C4′), 141.0 (C, C1′), 148.2 (C, C6a), 161.1 (C, C8a), 161.4 (C, C7a), 162.4 (C, C13). Anal. Calcd. for C_25_H_20_N_2_O_2_: C, 78.93; H, 5.30; N, 7.36. Found: C, 78.97; H, 5.27; N, 7.33.

*15-(4′-Methylphenyl)-10,11,12,15-tetrahydro-9H-benzo[5,6]chromeno[2,3-d]pyrido[1,2-a]pyrimidin-14-one* (**3Cb**): Yield 94%; mp ˃ 260 °C; IR (KBr) ν_max_ 1658, 1586 cm^−1^; ^1^H-NMR (CDCl_3_) 1.83–1.88 (m, H10, 2H), 1.90–1.95 (m, H11, 2H), 2.22 (s, CH_3_, 3H), 2.92–2.96 (m, H9, 2H), 3.90–3.97 (m, H12, 2H), 5.86 (s, H15, 1H), 7.03 (d, *J* = 7.6 Hz, H3′, H5′, 2H), 7.28–7.48 (m, H2, H3,H4,H5,H6, 5H), 7.81 (d, *J* = 8.1 Hz, H2′, H6′, 2H), 7.96 (d, *J* = 8.4 Hz, H1, 1H); ^13^C-NMR (CDCl_3_) 19.0 (CH_2_, C10), 21.0 (CH_3_), 21.7 (CH, C11), 31.4 (CH, C9), 36.2 (CH, C15), 43.0 (CH, C12), 101.1 (C, C14a), 116.7 (CH, C6), 117.5 (CH, C1), 123.7 (CH, C3), 124.9 (CH, C2), 127.1 (CH, C4), 128.4 (CH, C5), 128.9 (2CH, C2′, C6′), 129.1 (2CH, C3′, C5′), 129.2 (C, C15b), 131.1 (C, C15a), 131.4 (C, C4a), 136.2 (C, C4′), 141.0 (C, C1′), 148.1 (C, C6a), 158.5 (C, C8a), 159.0 (C, C7a), 162.3 (C, C14). Anal. Calcd. for C_26_H_22_N_2_O_2_: C, 79.17; H, 5.62; N, 7.10. Found: C, 79.12; H, 5.65; N, 7.14.

*16-(4’-Methylphenyl)-10,11,12,13-tetrahydro-16H-benzo[5,6]chromeno[2′,3′,4,5]pyrimido[1,2-a]azepin-15(9H)-one* (**3Cc**): Yield 86%; mp ˃ 260 °C; IR (KBr) ν_max_ 1659, 1589 cm^−1^; ^1^H-NMR (CDCl_3_) 1.82–2.04 (m, H9, H10, H11, H12, 8H), 2.22 (s, 3H, CH_3_, 3H), 4.05–4.11 (m, H13, 2H), 5.86 (s, H16, 1H), 7.03 (d, *J* = 8.7 Hz, H3′, H5′, 2H), 7.28–7.48 (m, H2, H3, H4, H5, H6, 5H), 7.80 (d, *J* = 9 Hz, H2′,H6′, 2H), 7.98 (d, *J* = 8.4 Hz, H1, 1H); ^13^C-NMR (CDCl_3_) 21.0 (CH, C11), 24.5 (CH_3_), 27.2 (CH_2_, C10), 29.6 (CH, C12), 36.5 (CH, C9), 37.3 (CH, C16), 43.1 (CH, C13), 101.3 (C, C15a), 116.9 (CH, C6), 117.5 (CH, C1), 123.0 (CH, C3), 123.7 (CH, C2), 124.8 (CH, C4), 127.0 (CH, C5), 128.4 (2CH, C2′, C6′), 129.0 (2CH, C3′, C5′), 129.1 (C, C16b), 131.1 (C, C16a), 131.5 (C, C4a), 136.1 (C, C4′), 141.0 (C, C1′), 148.1 (C, C6a), 159.3 (C, C8a), 162.1 (C, C7a), 163.3 (C, C15). Anal. Calcd. for C_27_H_24_N_2_O_2_: C, 79.39; H, 5.92; N, 6.86. Found: C, 79.41; H, 5.90; N, 6.89.

*7-Phenyl-11,12-dihydro-7H-benzo[7,8]chromeno[2,3-d]pyrrolo[1,2-a]pyrimidin-8(10H)-one* (**4Aa**): Yield 79%; mp ˃ 260 °C; IR (KBr) ν_max_ 1666, 1577 cm^−1^; ^1^H-NMR (CDCl_3_) 2.41–2.51 (m, 2H, H11), 3.07–3.17 (m, H12, 2H), 3.71–3.94 (m, H10, 2H), 5.31 (s, H7, 1H), 6.93–7.81 (m, H4, H2, H3, H5, H2′, H5′, H3′, H6′, H4′, H6, 10 H), 8.49 (d, *J* = 8.4 Hz, H1, 1H); ^13^C-NMR (CDCl_3_) 19.0 (CH_2_, C11), 32.5 (CH_2_, C12), 39.9 (CH, C7), 47.0 (CH_2_, C10), 100.2 (C, C7a), 118.6 (CH, C5), 121.6 (CH, C1), 123.8 (C, C6a), 124.7 (CH, C3), 126.4 (CH, C6), 126.5 (C, C14b), 126.8 (CH, C4′), 127.5 (CH, C2), 128.4 (CH, C4), 128.5 (2CH, C3′, C5′), 129.1 (2CH, C2′, C6′), 144.6 (C, C1′), 144.9 (C, C14a), 161.2 (C, C12a), 161.9 (C, C13a), 162.8 (C, C8). Anal. Calcd. for C_24_H_18_N_2_O_2_: C, 78.67; H, 4.95; N, 7.65. Found: C, 78.63; H, 4.97; N, 7.69.

*7-Phenyl-10,11,12,13-tetrahydro-7H-benzo[7,8]chromeno[2,3-d]pyrido[1,2-a]pyrimidin-8-one* (**4Ab**): Yield 90%; mp 259 °C; IR (KBr) ν_max_ 1667, 1572 cm^−1^; ^1^H-NMR (CDCl_3_) 1.92–1.94 (m, H10, 2H), 2.99–3.03 (m, H13, 2H), 3.88–3.91 (m, H11, H12, 4H), 5.33 (s, H7, 1H), 7.14–7.62 (m, H2, H3, H5, H2′, H5′, H3′, H6′, H4′, H6, 9H), 7.79 (d, *J* = 8.1 Hz, H4, 1H), 8.51 (d, *J* = 8.4 Hz, H1, 1H); ^13^C-NMR (CDCl_3_) 18.6 (CH_2_, C12), 21.3 (CH_2_, C11), 39.6 (CH, C7), 42.3 (CH_2_, C10), 45.2 (CH_2_, C13), 99.9 (C, C7a), 118.2 (CH, C5), 121.2 (CH, C1), 123.4 (C, C6a), 124.0 (CH, C3), 125.8 (CH, C6), 126.0 (C, C15b), 126.1 (CH, C4′), 126.2 (CH, C2), 127.0 (CH, C4), 127.9 (2CH, C2′, C6′), 128.1 (2CH, C3′, C5′), 132.7 (C, C4a), 144.1 (C, C1′), 144.5 (C, C15a), 158.2 (C, C13a), 162.0 (C, C14a), 177.3 (C, C8). Anal. Calcd. for C_25_H_20_N_2_O_2_: C, 78.93; H, 5.30; N, 7.36. Found: C, 78.97; H, 5.27; N, 7.32.

*7-Phenyl-11,12,13,14-tetrahydro-7H-benzo[7,8]chromeno[2′,3′,4,5]pyrimido[1,2-a]azepin-8(10H)-one* (**4Ac**): Yield 87%; mp 228 °C; IR (KBr) ν_max_ 1659, 1589 cm^−1^; ^1^H-NMR (CDCl_3_) 1.76–1.87 (m, H14,H13, H11, H12, 8H), 3.02–3.04 (m, H10, 2H), 5.34 (s, 1H, H7, 1H), 7.14–7.62 (m, H2, H3, H5, H2′, H5′, H3′, H6′, H4′, H6, 9H), 7.79 (d, *J* = 8.1 Hz, H4, 1H), 8.51 (d, *J* = 8.4 Hz, H1, 1H); ^13^C-NMR (CDCl_3_) 27.3 (CH, C13), 29.6 (CH_2_,C12), 30.5 (CH_2_, C11), 37.3 (CH_2_,C14), 40.4 (CH,C7), 43.0 (CH_2_,C10), 100.6 (C, C7a), 118.7 (CH, C5), 121.7 (CH, C1), 123.8 (C, C6a), 124.5 (CH, C3), 126.3 (CH, C6), 126.5 (C, C16b), 126.6 (CH, C4′), 126.7 (CH, C2), 127.5 (CH, C4), 128.4 (2CH, C3′, C5′), 128.5 (2CH, C2′, C6′), 133.2 (C, C4a), 144.5(C, C1′), 145.0 (C, C16a), 159.8 (C, C14a), 162.2 (C, C15a), 163.6 (C, C8). Anal. Calcd. for C_26_H_22_N_2_O_2_: C, 79.17; H, 5.62; N, 7.10. Found: C, 79.12; H, 5.64; N, 7.15.

*7-(3′-Methoxyphenyl)-11,12-dihydro-7H-benzo[7,8]chromeno[2,3-d]pyrrolo[1,2-a]pyrimidin-8(10H)-one* (**4Ba**): Yield 81%; mp 224 °C; IR (KBr) ν_max_ 1663, 1577 cm^−1^; ^1^H-NMR (CDCl_3_) 2.22–2.27 (m, H11, 2H), 3.18–3.20 (m, H12, 2H), 3.74 (s, OCH_3_, 3H), 4.09–4.16 (m, H10, 2H), 5.36 (s, H7, 1H), 6.71 (d, *J* = 7.8 Hz, H6, 1H), 6.91–7.60 (m, H2, H3, H5, H2′, H5′, H6′, H4′, 7H), 7.81 (d, *J* = 7.8 Hz, H4, 1H), 8.49 (d, *J* = 8.4 Hz, H1, 1H); ^13^C-NMR (CDCl_3_) 19.1 (CH, C11), 32.5 (CH, C12), 39.9 (CH, C7), 47.0 (CH, C10), 55.1 (OCH_3_), 100.7 (C, C7a), 111.9 (CH, C4′), 114.1 (CH, C2′), 118.4 (CH, C5), 120.9 (CH, C6′), 121.6 (C, C6a), 123.8 (CH, C1), 124.7 (C, C14b), 126.4 (CH, C3), 126.6 (2CH, C2, C6), 127.5 (CH, C4), 129.3 (CH, C5′), 133.3 (C, C4a), 144.5 (C, C1′), 146.5 (C, C14a), 159.6 (C, C12a), 161.1 (C, C13a), 161.8 (C, C8), 162.8 (C, C3′). Anal. Calcd. for C_25_H_20_N_2_O_3_: C, 75.74; H, 5.09; N, 7.07. Found: C, 75.72; H, 5.11; N, 7.12.

*7-(3′-Methoxyphenyl)-10,11,12,13-tetrahydro-7H-benzo[7,8]chromeno[2,3-d]pyrido[1,2-a]pyrimidin-8-one* (**4Bb**): Yield 90%; mp 238 °C; IR (KBr) ν_max_ 1661, 1572 cm^−1^; ^1^H-NMR (CDCl_3_)1.89–1.94 (m, 4H, H11, H12, 4H), 2.88–2.95 (m, H13, 2H), 3.71 (s, OCH_3_, 3H), 3.87–3.89 (m, H10, 2H), 5.31 (s, H7, 1H), 6.69 (d, *J* = 8.1 Hz, H6, 1H), 6.89–7.59 (m, H2, H3, H5, H2′, H5′, H6′, H4′, 7H), 7.76 (d, *J* = 7.8 Hz, H4, 1H), 8.48 (d, *J* = 8.4 Hz, H1, 1H); ^13^C-NMR (CDCl_3_) 18.6 (CH, C12), 21.3 (CH, C11), 31.1 (CH,C13), 39.6 (CH, C7), 42.4 (CH, C10), 54.6 (OCH_3_), 99.7 (C, C7a), 111.3 (CH, C4′), 114.1 (CH, C2′), 118.1 (CH, C5), 120.5 (CH, C6′), 121.2 (C, C6a), 123.4 (CH, C1), 124.0 (C, C15b), 125.8 (CH, C3), 126.0 (2CH, C2, C6), 127.0 (CH, C4), 128.8 (CH, C5′), 132.7 (C, C4a), 143.9 (C, C1′), 146.2 (C, C15a), 158.2 (C, C13a), 159.1 (C, C14a), 159.4 (C, C8), 162.0 (C, C3′). Anal. Calcd. for C_26_H_22_N_2_O_3_: C, 76.08; H, 5.40; N, 6.82. Found: C, 76.12; H, 5.37; N, 6.79.

*7-(3′-Methoxyphenyl)-11,12,13,14-tetrahydro-7H-benzo[7,8]chromeno[2′,3′,4,5]pyrimido[1,2-a]azepin-8(10H)-one* (**4Bc**): Yield 79%; mp 215 °C; IR (KBr) ν_max_ 1662, 1577 cm^−1^; ^1^H-NMR (CDCl_3_) 1.80–2.11 (m, H14, H11, H13, H12, 8H), 3.10–3.17 (m, H10, 2H), 4.10 (s, OCH_3_, 3H), 5.29 (s, H7, 1H), 6.70 (d, *J* = 7.8 Hz, H6, 1H), 6.89–7.62 (m, H2, H3, H5, H2′, H5′, H6′, H4′, 7H), 7.79 (d, *J* = 7.8 Hz, H4, 1H), 8.51 (d, *J* = 8.1 Hz, H1, 1H); ^13^C-NMR (CDCl_3_) 24.0 (CH, C12), 26.7 (CH, C13), 29.1 (CH, C11), 36.4 (CH, C14), 39.8(CH, C7), 42.6 (CH, C10), 54.6 (OCH_3_), 100.0 (C, C7a), 111.4 (CH, C4′), 114.1 (CH, C2′), 118.0 (CH, C5), 120.5 (CH, C6′), 121.2 (C, C6a), 123.3 (CH, C1), 124.1 (C, C16b), 125.2 (CH, C3), 125.9 (2CH, C2,C6), 126.1 (CH, C4), 126.9 (CH, C5′), 128.8 (C, C4a), 132.7 (C, C1′), 143.8 (C, C16a), 146.0 (C, C14a), 159.1 (C, C15a), 161.4 (C, C8), 163.3 (C, C3′). Anal. Calcd. for C_27_H_24_N_2_O_3_: C, 76.40; H, 5.70; N, 6.60. Found: C, 76.37; H, 5.72; N, 6.63.

*7-(4′-Methylphenyl)-11,12-dihydro-7H-benzo[7,8]chromeno[2,3-d]pyrrolo[1,2-a]pyrimidin-8(10H)-one* (**4Ca**): Yield 78%; mp ˃ 260 °C; IR (KBr) ν_max_ 1661, 1589 cm^−1^; ^1^H-NMR (CDCl_3_) 2.14–2.21 (m, H11, 2H), 2.27 (s, CH_3_, 3H), 3.11–3.22 (m,H12, 2H), 4.05–4.12 (m, H10, 2H), 5.35 (s, H7, 1H), 7.06 (d, *J* = 7.8 Hz, H3′, H5′, 2H), 7.17 (d, *J* = 8.7 Hz, H6, 1H), 7.25 (d, *J*= 7.8 Hz, H2′, H6′, 2H), 7.51–7.62 (m, H2, H3, H5, 3H), 7.72 (d, *J* = 8.4 Hz, H4, 1H) 8.49 (d, *J* = 8.4 Hz, H1, 1H); ^13^C-NMR (CDCl_3_) 19.11 (CH_2_, C11), 21.0 (CH_3_), 32.5 (CH_2_, C12), 39.5 (CH, C7), 46.9 (CH_2_, C10), 101.0 (C, C7a), 118.7 (CH, C5), 121.5 (CH, C1), 124.6 (C, C6a), 126.5 (CH, C3), 126.6 (CH, C6), 127.1 (CH, C2), 127.5 (C, C14b), 128.4 (CH, C4), 129.1 (2CH, C2′, C6′), 129.8 (2CH, C3′, C5′), 133.2 (C, C4a), 136.4 (C, C4′), 142.1 (C, C1′), 144.5 (C, C14a), 161.2 (C, C12a), 161.8 (C, C13a), 162.7 (C, C8). Anal. Calcd. for C_25_H_20_N_2_O_2_: C, 78.93; H, 5.30; N, 7.36. Found: C, 78.97; H, 5.27; N, 7.38.

*7-(4′-Methylphenyl)-10,11,12,13-tetrahydro-7H-benzo[7,8]chromeno[2,3-d]pyrido[1,2-a]pyrimidin-8-one* (**4Cb**): Yield 86%; mp 224 °C; IR (KBr) ν_max_ 1667, 1571 cm^−1^; ^1^H-NMR (CDCl_3_) 1.92–1.99 (m, H11,H12, 4H), 2.27 (s, CH_3_, 3H), 3.02–3.10 (m, H13, 2H), 3.90–3.96 (m, H10, 2H), 5.29 (s, H7, 1H), 7.06 (d, *J* = 7.8 Hz, H3′, H5′, 2H), 7.14 (d, *J* = 8.1 Hz, H6, 1H), 7.23 (d, *J* = 7.8 Hz, H2′, H6′, 2H), 7.52–7.63 (m, H2, H3, H5, 3H), 7.79 (d, *J* = 7.8 Hz, H4, 1H), 8.52 (d, *J* = 8.4 Hz, H1, 1H); ^13^C-NMR (CDCl_3_) 18.9 (CH_2_, C12), 21.0 (CH_3_), 21.7 (CH_2_, C11), 31.2 (CH_2_, C13), 39.6 (CH, C7), 42.9 (CH_2_, C10), 100.6 (C, C7a), 118.7 (CH, C5), 123 (CH, C1), 124.7 (C, C6a), 126.5 (2CH, C3, C6), 127.0 (CH, C2), 127.5 (C, C15b), 127.8 (CH, C4), 128.4 (2CH, C2′, C6′), 129.1 (2CH, C3′, C5′), 133.2 (C, C4a), 136.4 (C, C4′), 141.9 (C, C1′), 144.3 (C, C15a), 158.9 (C, C13a), 159.1 (C, C14a), 162.1 (C, C8). Anal. Calcd. for C_26_H_22_N_2_O_2_: C, 79.17; H, 5.62; N, 7.10. Found: C, 79.21; H, 5.59; N, 7.14.

*17-(4′-Methylphenyl)-11,12,13,14-tetrahydro-7H-benzo[7,8]chromeno[2′,3′,4,5]pyrimido[1,2-a]azepin-8(10H)-one* (**4Cc**): Yield 70%; mp > 260 °C; IR (KBr) ν_max_ 1664, 1593 cm^−1^; ^1^H-NMR (CDCl_3_) 1.74–1.81 (m, H12, 2H), 1.85–1.96 (m, H11, H13, H14, 6H), 2.27 (s, CH_3_, 3H), 3.02–3.11 (m, H10, 2H), 5.30 (s, H7, 1H), 7.06 (d, *J* = 7.8 Hz, H3′, H5, 2H′), 7.14 (d, *J* = 8.1 Hz, H6, 1H), 7.24 (d, *J* = 7.8 Hz, H2′, H6′, 2H), 7.50–7.62 (m, H2, H3, H5, 3H), 7.70 (d, *J* = 7.8 Hz, H4, 1H), 7.79 (d, *J* = 8 Hz, H1, 1H); ^13^C-NMR (CDCl_3_) 21.0 (CH_3_), 24.6 (CH_2_, C12), 27.3 (CH_2_, C13), 29.6 (CH_2_,C11), 37.3 (CH_2_, C14), 40.0 (CH, C7), 43.0 (CH_2_, C10), 100.7 (C, C7a), 118.9 (CH, C5), 121.7 (CH, C1), 123.9 (C, C6a), 124.5 (CH, C3), 126.6 (CH, C6), 127.2 (C, C16b), 127.8 (CH, C4), 128.4 (2CH, C2′, C6′), 129.1 (2CH, C3′,C5′), 133.2 (C,C4a), 136.3 (C, C4′), 142.2 (C, C1′), 144.4 (C, C16a), 159.7 (C, C14a), 162.1 (C, C15a), 163.5 (C, C8). Anal. Calcd. For C_27_H_24_N_2_O_2_: C, 79.39; H, 5.92; N, 6.86. Found: C, 79.35; H, 5.94; N, 6.89.

### 3.2. Oxygen Radical Absorbance Capacity Assay

The antioxidant power of BCPOs was determined following the ORAC-FL method using fluorescein as the fluorescent probe [20,21]. (±)-6-Hydroxy-2,5,7,8-tetramethylchromane-2-carboxylic acid (Trolox), fluorescein (FL) and AAPH were bought from Sigma-Aldrich (Saint Louis, MO, USA). A Varioskan Flash plate reader with built-in injectors (Thermo Scientific, Waltham, MA, USA) was used. The final volume reaction mixture was 200 µL and the reaction was carried out at 37 °C in 75 mM phosphate buffer (pH 7.4). The tested compounds and Trolox standard were dissolved in DMSO to 10 mM and further diluted in phosphate buffer. The final concentrations were 0.1–1 µM for the tested compounds and 1–8 µM for Trolox standard. The blank was composed of 120 µL of FL, 60 µL of AAPH and 20 µL of phosphate buffer (pH = 7.4) and was added in each assay. The antioxidant (20 µL) and fluorescein (FL, 120 µL, final concentration of 70 nM) were incubated in a black 96-well microplate Nunc, purchazed from Thermo Fisher Scientific, Ecublens, Switzerland) for 15 min at 37 °C. Then, AAPH, 60 µL, final concentration of 12 mM) solution was added quickly using the built-in injector. The fluorescence decay was measured every minute for 60 min at λ_ex_ = 485 nm and λ_em_ = 535 nm. The microplate was automatically shaken prior to each reading. All the experiments were made in triplicate and at least three different assays were performed for each sample. Antioxidant curves (fluorescence versus time) were first normalized to the curve of the blank (without antioxidant) and then, the area under the fluorescence decay curve (AUC) was calculated as:

AUC = 1 + sum (*f*_i_/*f*_0_) (1)

where *f*_0_ is the initial fluorescence reading at 0 min and *f_i_* is the fluorescence value at time *i*. The net AUC corresponding to the sample was calculated as follows:

Net AUC = AUC_antioxidant_ − AUC_blank_ (2)

Using MS Excel software, regression equations were extrapolated by plotting the net AUC against the antioxidant concentration. The ORAC values were then obtained by dividing the slope of the latter curve between the slope of the Trolox curve obtained in the same assay. Final ORAC values were expressed as Trolox equivalents. Data are expressed as mean ± SD.

### 3.3. Inhibition of EeAChE and EqBuChE

Assessment of the inhibitory power of BCPOs was performed following the spectrophotometric method of Ellman [23] using purified AChE from *Electrophorus electricus* (Type V-S, Sigma-Aldrich) or BuChE from horse serum (lyophilized powder, Sigma-Aldrich). Enzymes were first dissolved in 0.1 M phosphate buffer (pH 8.0) and then aliquoted in small vials for easy handling. Compounds stock solutions in DMSO (10 mM) were diluted when necessary with DMSO to prepare appropriate dilutions of each compound. The assay was performed in a final volume of 3 mL of a 0.1 M phosphate-buffered solution at pH 8.0, containing 5,5′-dithiobis-2-nitrobenzoic acid (DTNB, 2625 µL, 0.35 mM, final concentration), *Ee*AChE (29 µL, 0.035 U/mL final concentration) or *eq*BuChE (60 µL, 0.05 U/mL final concentration) tested compound (3 µL, different concentrations) and 1% (*w*/*v*) Bovine Albumin Serum phosphate-buffered (pH 8.0) solution (BSA, 40 µL). The inhibition in comparison to control without compound was determined by pre-incubating this blend at room temperature with each compound at nine different concentrations for 10 min. Then, acetylthiocholine iodide (105 µL, 0.35 mM, final concentration) or butyrylthiocholine iodide (150 µL, 0.5 mM final concentration) was added and incubated for another 15 min at rt. The absorbances were measured at 412 nm in a plate reader (iEMS Reader MF, Labsystems, Thermo fisher scientific Ecublens, Switzerland). The percentage of inhibition of the enzyme was calculated in comparison with the blank sample (100% enzyme activity). Calculation of IC_50_ values was performed with GraphPad Prism 5. Each concentration was measured in triplicate. Data are expressed as mean ± SEM.

### 3.4. Inhibition of hAChE

Ellman’s assay was followed to evaluate the anticholinesterasic potency of BCPOs [23] using human recombinant AChE (Sigma-Aldrich). 500 U of *h*AChE were dissolved in 1 mL of a gelatine solution (1% in water) and diluted with demineralized water to give a stock solution of 5 U/mL. The 12.5 mM 5,5′-dithiobis-(2-nitrobenzoic acid) (DTNB, Ellman’s reagent) solution containing 0.15% (*w*/*v*) sodium carbonate and 18.75 mM acetylthiocholine (ATC) iodide solution were prepared in demineralized water. All assays were performed in 0.1 M phosphate buffer pH 8.0. The assayed compounds or blank (water) (25 µL) were incubated with the enzyme (20 µL) for 5 min at 37 °C in 765 µL of phosphate buffer prior to start the reaction. Then, 20 µL of DTNB and 20 µL of ATC were added. After 5 min, absorbances were measured at 412 nm with an EnSpire Multimode microplate reader (Perkin-Elmer, Waltham, MA, USA). The percentage of inhibition of the enzyme was calculated by comparison with a blank sample (100% enzyme activity). IC_50_ values were determined with GraphPad Prism 5. Each concentration was measured in triplicate. Data are expressed as mean ± SEM.

### 3.5. Kinetic Characterization of hAChE Inhibition

To estimate the type of inhibition of *h*AChE, we performed the same experimental protocol as reported for *h*AChE inhibition. Different concentrations of the substrate ATC (0.067–0.5 mM) were used to create Lineweaver-Burk plots by plotting the inverse initial velocity (1/*V*) as a function of the inverse of the substrate concentration (1/[S]). The stock solution of ATC (0.5 mM in a well) was prepared in demineralized water and diluted before use to obtain 0.4, 0.3, 0.2, 0.1 and 0.067 mM substrate solutions. The double reciprocal plots were analysed by a weighted least square procedure that assumed the variance of *V* to be constant. Each experiment was performed in triplicate. To confirm the mode of inhibition, Cornish-Bowden plots were obtained by plotting S/*V* (substrate/velocity ratio) versus the inhibitor concentration [23,28]. Data analysis was performed with GraphPad Prism 5. Inhibition constant (K_i_) values were determined by re-plotting slopes from the Lineweaver-Burk plot versus the inhibitor concentration where K_i_ was determined as the intersect of the line with the x-axis [29].

### 3.6. In vitro Toxicity of Compounds ***3Ab**, 3**Bb**, **3Cb*** and ***3Ba*** in HepG2 Cells

HepG2 cells were purchased from American Type Culture Collection. The cells were cultured in Eagle’s Minimum Essential Medium (Ozyme, France) supplemented with 10% fetal bovine serum, 1X non-essential amino acids, 100 units/mL penicillin and 10 mg/mL streptomycin (Dutscher, Brumath, France). Cultures were kept under a CO_2_/air (5%/95%) humidified atmosphere at 37 °C. Prior to the experiment, cells were seeded in 96-well culture plates at a density of 0.1 × 10^6^ cells per well. After 24 h of incubation, the culture medium was refreshed and 100 µL of the test compounds or DMSO (0.1%) were added. Compounds were tested at 4 concentrations (1–30 µM) in triplicate. For the MTT assay [25], after 24 h of treatment, cells were incubated with 50 µL MTT (0.5 mg/mL, Sigma Aldrich, city, France) at 37 °C for 2 h. Plates were centrifuged, MTT was removed and 100 µL DMSO was distributed per well. The absorbance at 570 nm was measured using microplate reader (brand). Cell viability was expressed as percentage of cell viability compared to controls (DMSO, 0.1%).

### 3.7. PAMPA Assay

Penetration across the BBB is an essential property for compounds targeting the CNS. In order to predict passive blood-brain penetration of novel compounds modification of the PAMPA has been used based on reported protocol [26,27]. The filter membrane of the donor plate was coated with PBL (Polar Brain Lipid, Avanti, AL, USA) in dodecane (4 µL of 20 mg/mL PBL in dodecane) and the acceptor well was filled with 300 µL of PBS pH 7.4 buffer (V_D_). Tested compounds were dissolved first in DMSO and that diluted with PBS pH 7.4 to reach the final concentration 100 µM in the donor well. Concentration of DMSO did not exceed 0.5% (*v*/*v*) in the donor solution. 300 µL of the donor solution was added to the donor wells (V_A_) and the donor filter plate was carefully put on the acceptor plate so that coated membrane was “in touch” with both donor solution and acceptor buffer. Test compound diffused from the donor well through the lipid membrane (Area = 0.28 cm^2^) to the acceptor well. The concentration of the drug in both donor and the acceptor wells was assessed after 3, 4, 5 and 6 h of incubation in quadruplicate using the UV plate reader Synergy HT (Biotek, Winooski, VT, USA) at the maximum absorption wavelength of each compound. Concentration of the compounds was calculated from the standard curve and expressed as the permeability (Pe) according the Equation (1) [30,31]:

(3) $$\log P_{e}=\log\;\left\{C\times -ln\left(1-\frac{{\left[drug\right]}_{acceptor}}{{\left[drug\right]}_{equilibrium}}\right)\right\}\;where\;C=\;\left(\frac{V_{D}\times V_{A}}{\left(V_{D}+V_{A}\right)\times Area\times time}\right)$$

## 4. Conclusions

We have synthesized and evaluated eighteen new benzochromenopyrimidinones as promising multitarget-directed ligands with marked selectivity for AChE and good antioxidant activity. Particularly, compounds **3Ab**, **3Bb**, **3Cb** and **3Ba** were found to be non-hepatotoxic and moderate *h*AChEIs. Among them, although compound **3Bb** showed a Pe value in an uncertain interval and consequently, a compromised permeability, this benzochromenopyrimidinone is a micromolar mixed-type *h*AChE inhibitor (IC_50_ = 1.28 μM) and a potent antioxidant (4.7 TE). To sum up, this small library of benzochromenopyrimidinones constitutes an additional step in our laboratory towards the search for lead compounds with polypharmacological properties as potential new anti-AD agents.

## Figures and Tables

**Table 1:** Inhibitions of *Ee*AChE, *eq*BuChE, *h*AChE and ORAC-FL values for compounds **3** and **4**. 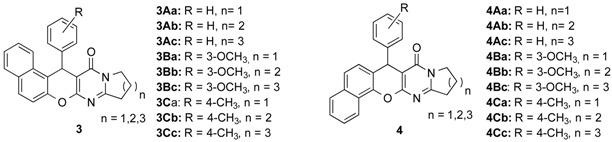

BCPO	*Ee*AChE ^1^ (IC_50_, nM)	*eq*BuChE (% Inhibition at 10 µM)	*h*AChE (IC_50_, nM)	ORAC ^2^
**3Aa**	518.4 ± 87.9	12.1 ± 1.4	n.d.	2.6 ± 0.1
**3Ab**	55.5 ± 7.1	31.2 ± 1.8	3657 ± 59	2.3 ± 0.3
**3Ac**	300.8 ± 6.5	28.4 ± 7.3	n.d.	2.5 ± 0.2
**3Ba**	60.7 ± 4.5	51.0 ± 4.0	1527 ± 25	3.4 ± 0.2
**3Bb**	30.5 ± 2.8	43.0 ± 1.9	1279 ± 32	4.7 ± 0.2
**3Bc**	107.5 ± 7.2	n.a.	n.d.	3.5 ± 0.3
**3Ca**	111.9 ± 21.7	17.4 ± 1.8	n.d.	2.7 ± 0.3
**3Cb**	55.9 ± 12.7	19.0 ± 2.0	1591 ± 24	3.9 ± 0.3
**3Cc**	166.6 ± 7.8	n.a.	n.d.	3.1 ± 0.2
**4Aa**	317.8 ± 26.0	23.1 ± 3.1	n.d.	2.1 ± 0.1
**4Ab**	383.5 ± 19.4	54.6 ± 1.8	n.d.	2.3 ± 0.2
**4Ac**	290.5 ± 8.3	24.2 ± 3.1	n.d.	2.5 ± 0.1
**4Ba**	326.7 ± 38.9	38.0 ± 1.6	n.d.	3.8 ± 0.1
**4Bb**	153.2 ± 3.1	n.a.	n.d.	3.7 ± 0.2
**4Bc**	195.3 ± 6.2	31.1 ± 2.1	n.d.	3.4 ± 0.2
**4Ca**	115.8 ± 6.2	26.8 ± 2.9	n.d.	3.2 ± 0.1
**4Cb**	193.4 ± 18.7	24.4 ± 1.2	n.d.	2.9 ± 0.1
**4Cc**	173.8 ± 5.9	n.a.	n.d.	3.6 ± 0.2
**Tacrine**	44.3 ± 1.5 [19]	IC_50_ = 5.1 ± 0.2 nM [19]	131 ± 2	0.2 ± 0.1 [22]
**Ia**	82.5 M [24]	IC_50_ = 25.1 [24]	n.d.	n.d.
**Ib**	38.6 M [24]	>500 [24]	n.d.	n.d.
**Ic**	279 M [24]	>500 [24]	n.d.	n.d.
**II**	6.3 M [15]	IC_50_ = 8.4 M [15]	n.d.	n.d.
Ferulic acid	n.d.	n.d.	n.d.	3.7 ± 0.1 [22]

^1^ IC_50_ values were obtained by nonlinear regression. *Ee*: electric eel, *eq*: serum horse, *h*: human. Each IC_50_ value is the mean ± SEM of at least three independent experiments. ^2^ Data are expressed as Trolox equivalents and are shown as mean ± SD. n.a.: Not active (any inhibition was observed). n.d.: not determined.

**Table 2:** *In vitro* toxicity (% cell viability) of selected **3Ab**, **3Bb**, **3Cb** and **3Ba** and tacrine in HepG2 cells.

BCPO	1 µM	3 µM	10 µM	30 µM	100 µM	300 µM	1 mM
**3Ab**	99.3 ± 4.0	95.3 ± 2.3	98.5 ± 2.5	91.5 ± 4.5	100.8 ± 3.1	110.5 ± 5.5	110.4 ± 4.4
**3Bb**	107.7 ± 2.4	111.1 ± 8.7	104.7 ± 4.2	104.1 ± 5.0	111.8 ± 2.3	118.0 ± 7.3	127.8 ± 5.0
**3Cb**	105.8 ± 7.0	102.9 ± 10.8	110.2 ± 9.1	112.7 ± 5.8	103.1 ± 5.7	113.0 ± 5.2	110.2 ± 8.0
**3Ba**	108.6 ± 2.0	107.7 ± 4.8	106.2 ± 3.5	99.2 ± 4.2	97.0 ± 5.4	107.6 ± 2.8	119.5 ± 6.8
**Tacrine**	105.2 ± 4.6	103.5 ± 8.7	97.0 ± 6.5	93.0 ± 2.7	95.2 ± 6.0	47.6 ± 5.6 ***	13.9 ± 0.8 ***

Means ± SEM of triplicates from at least three different cultures. *** *p* < 0.001, as compared to the control cultures (one-way ANOVA).

**Table 3:** Prediction of BBB penetration of drugs expressed as Pe ± SEM (*n* = 6–8).

Compound	BBB Penetration Estimation	
	Pe ± SEM (×10^−6^ cm s^−1^)	CNS (+/−)
**3Ab**	7.2 ± 0.6	CNS (+)
**3Bb**	3.6 ± 0.57	CNS (+/−)
**3Cb**	ND *	
**3Ba**	4.6 ± 0.77	CNS (+)
Donepezil	7.3 ± 0.9	CNS (+)
Rivastigmine	6.6 ± 0.5	CNS (+)
Tacrine	5.3 ± 0.19	CNS (+)
Testosterone	11.3 ± 1.6	CNS (+)
Chlorpromazine	5.6 ± 0.6	CNS (+)
Hydrocortisone	2.85 ± 0.1	CNS (+/−)
Piroxicam	2.2 ± 0.15	CNS (+/−)
Theophyline	1.07 ± 0.18	CNS (−)
Atenolol	1.02 ± 0.37	CNS (−)

‘CNS (+)’ (high BBB permeability predicted); Pe (10^−6^ cm·s^−1^) > 4.0. ‘CNS (−) (low BBB permeability predicted); Pe (10^−6^ cm·s^−1^) < 2.0. ‘CNS (+/−) (BBB permeability uncertain); Pe (10^−6^ cm·s^−1^) from 4.0 to 2.0. * Not Determined due to a low solubility; CNS (+)= 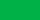; CNS (+/−)= 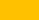; CNS (−)= 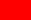.

## Supplementary Materials

Supplementary materials can be accessed at: http://www.mdpi.com/1420-3049/21/5/634/s1.
