# Supplementary material for: Synthesis and Biological Evaluation of Benzochromenopyrimidinones as Cholinesterase Inhibitors and Potent Antioxidant, Non-Hepatotoxic Agents for Alzheimer’s Disease
Source: Molecules. 2016 May 14;21(5):634. doi: 10.3390/molecules21050634 (PMC6273488; doi:10.3390/molecules21050634)
Supplement: Supplementary file 1 [file molecules-21-00634-s001.pdf]

Supplementary Materials: Synthesis and Biological Evaluation of Benzochromenopyrimidinones as Cholinesterase Inhibitors, and Potent Antioxidant, Non-Hepatotoxic Agents for Alzheimer's Disease

Youssef Dgachi, Oscar M. Bautista-Aguilera, Mohamed Benchekroun, Hélène Martin, Alexandre Bonet, Damijan Knez, Justyna Godyń, Barbara Malawska, Stanislav Gobec, Mourad Chioua, Jana Janockova, Ondrej Soukup, Fakher Chabchoub, José Marco-Contelles and Lhassane Ismaili

1. Inhibition of Aβ_{1-42} Aggregation

Table S1. Inhibition of Aβ_{1-42} aggregation.

Compound	Structure	Inhibition of Aβ_{1-42} Aggregation at 10 μM (%) [a]
**3Ab**	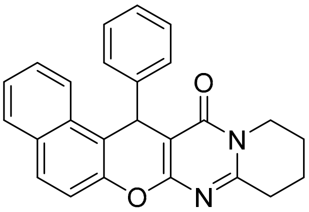	13.6 ± 2.1 *
**3Bb**	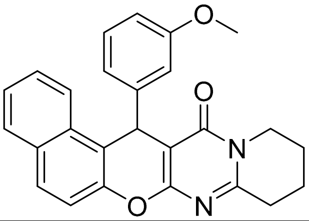	n.a. [b]
**3Cb** [c]	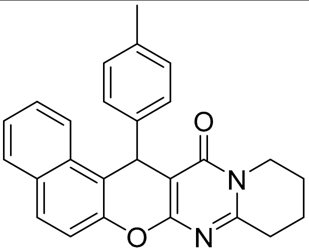	n.a. [b]
**3Ba**	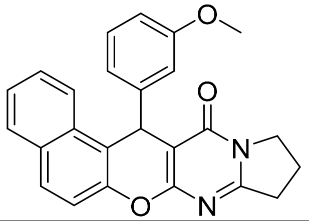	n.a. [b]

[a] Percent of inhibition at 10 μM compound and 1.5 μM Aβ_{1-42}. Percentage of inhibition is expressed as mean ± standard deviation of two independent experiments. * $p < 0.05$, compared to the control (one-way ANOVA, followed by Bonferoni *t*-test). [b] n.a.; inhibition lower than 10%, compound defined as not active. [c] poor solubility in DMSO at 10 mM and 1 mM.

Compound **3Ab** showed weak inhibition of Aβ_{1-42} aggregation, whereas other compound were not active.

2. Thioflavin-T (ThT) Fluorometric Assay [1]

Recombinant human 1,1,1,3,3,3-hexafluoro-2-propanol pretreated A$\beta_{1\text{–}42}$ peptide (Merck Millipore, Darmstadt, Germany) was dissolved in DMSO to give 75 μM stock solution. The stock solution was further diluted in HEPES buffered solution (150 mM HEPES, pH 7.4, 150 mM NaCl), to 7.5 μM. A$\beta_{1\text{–}42}$ solution was then added to the test compounds in black-walled 96-well plate, and diluted with ThT solution (final concentration of ThT was 10 μM). Final mixture contained 1.5 μM A$\beta_{1\text{–}42}$, 10 μM of test compounds, and 3% DMSO. ThT fluorescence was measured every 5 min (λ_{ex} = 440 nm, λ_{em} = 490 nm), with the medium continuously shaking between measurements using a 96-well microplate reader (Synergy™ H4, BioTek Instruments, Inc., Winooski, VT, USA). The fluorescence intensities at the plateau reached after 24 h in the absence and presence of the test compound were averaged, and the average fluorescence of the corresponding wells at t = 15 min was subtracted. The A$\beta_{1\text{–}42}$ aggregation inhibitory potency is expressed as the percentage inhibition (% inh = $(1 - F_i/F_0) \times 100\%$), where F_i is the increase in fluorescence of A$\beta_{1\text{–}42}$ treated with the test compound, and F_0 is the increase in fluorescence of A$\beta_{1\text{–}42}$ alone.

Reference

1. LeVine, H. Thioflavine T interaction with synthetic Alzheimer's disease beta-amyloid peptides: Detection of amyloid aggregation in solution. *Protein Sci. Publ. Protein Soc.* **1993**, *2*, 404–410.
